# The adaptive potential of subtropical rainbowfish in the face of climate change: heritability and heritable plasticity for the expression of candidate genes

**DOI:** 10.1111/eva.12363

**Published:** 2016-02-18

**Authors:** R. J. Scott McCairns, Steve Smith, Minami Sasaki, Louis Bernatchez, Luciano B. Beheregaray

**Affiliations:** ^1^ Molecular Ecology LaboratoryFlinders UniversityAdelaideSAAustralia; ^2^ Centre National de la Recherche ScientifiqueUMR 7263 (IMBE) Institut Méditerranéen de la Biodiversité et d'Ecologie Marine et ContinentaleMarseilleFrance; ^3^ Department of Integrative Biology and EvolutionUniversity of Veterinary MedicineViennaAustria; ^4^ Institut de Biologie Intégrative et des SystèmesUniversité LavalQuébecQCCanada

**Keywords:** adaptation, Australia, climate change, ecological genomics, evolutionary physiology, gene expression, teleost, thermal tolerance

## Abstract

Whilst adaptation and phenotypic plasticity might buffer species against habitat degradation associated with global climate change, few studies making such claims also possess the necessary and sufficient data to support them. Doing so requires demonstration of heritable variation in traits affecting fitness under new environmental conditions. We address this issue using an emerging aquatic system to study adaptation to climate change, the crimson‐spotted rainbowfish (*Melanotaenia duboulayi*), a freshwater species from a region of eastern Australia projected to be affected by marked temperature increases. Captive born *M. duboulayi* of known pedigree were used to assess the long‐term effects of contemporary and 2070‐projected summer temperatures on the expression of genes previously identified in a climate change transcriptomics (RNA‐Seq) experiment. Nearly all genes responded to increasing temperature. Significant additive genetic variance explained a moderate proportion of transcriptional variation for all genes. Most genes also showed broad‐sense genetic variation in transcriptional plasticity. Additionally, molecular pathways of candidate genes co‐occur with genes inferred to be under climate‐mediated selection in wild *M. duboulayi* populations. Together, these results indicate the presence of existing variation in important physiological traits, and the potential for adaptive responses to a changing thermal environment.

## Introduction

Global climate change poses many threats to biodiversity, but for ectotherms, the associated impacts affecting temperature niche may be particularly problematic (Pearson et al. 2014). Given pronounced intracontinental temperature increases (Diffenbaugh and Field 2013), and the influence of localized climatic effects on their thermal properties (Caissie 2006), inland and coastal freshwaters may be especially vulnerable to temperature increases. Indeed, freshwater habitats and their biota are among the most threatened by environmental change (Dudgeon et al. 2006), a concern highlighted in the most recent Intergovernmental Panel on Climate Change (IPCC) report (Settele et al. 2014). All freshwater fishes are ectotherms, and thus, metabolically sensitive to environmental temperature. How they are likely to respond to the challenges associated with global climate change is in many ways dependent upon the effects of new environmental inputs on essential physiological processes (Helmuth 2009; Chown et al. 2010; Hofmann and Todgham 2010). Within the context of population‐level responses to global climate change, adaptation and phenotypic plasticity are often cited as potential means of species persistence. Yet, few studies present empirical data essential to understanding whether either of these represents viable mechanisms. Critically absent from the discussion are demonstrations of a genetic basis for traits likely to influence fitness in the changed environments (Gienapp et al. 2008; Charmantier and Gienapp 2014; Merilä and Hendry 2014). Moreover, despite a perceived ubiquity of biological adaptation in nature, evidence of the potential to genetically adapt to climate change is scarce and ambiguous (Bradshaw and Holzapfel 2006; Hoffmann and Sgrò 2011; Merilä and Hendry 2014). This is particularly so in aquatic organisms from the southern hemisphere, which are largely under‐represented in climate change research (Chambers et al. 2013).

Acclimatization – acclimation within the context of a controlled, laboratory setting – is a compensatory change in physiological processes in response to environmental change (Willmer et al. 2005) and represents a form of phenotypic plasticity. Transcriptional mechanisms appear to play a key role in acclimation to environmentally induced stressors across broad taxonomic groups (López‐Maury et al. 2008), and in the regulation of metabolism (Desvergne et al. 2006). For ectothermic species such as fishes, the latter is also highly relevant with respect to changes in the thermal environment. In many fish species, acclimation can increase threshold temperatures for induction of the cellular stress response and upper thermal tolerance (Fangue et al. 2011; Currie and Schulte 2014). However, this may come at the cost of increased resting metabolic rate and related long‐term fitness consequences (Pörtner et al. 2006; Pörtner and Knust 2007; Muñoz et al. 2012). Conversely, interindividual variation in the thermal response may be reflective of genetic variability in the underlying physiological processes. Yet, whilst a genetic basis to functional biology is frequently assumed in the literature, estimates of heritability for variation in physiological processes are scarce, especially in fishes (Chown et al. 2010). This is particularly problematic with respect to forecasting the effects of climate change, as any adaptive response is dependent upon the degree of additive genetic variation underlying organisms’ thermal physiology. If we are to address these issues in a rigorous manner, we require ecologically relevant models that are also amenable to multigenerational experimentation.

Rainbowfishes (Melanotaeniidae) are a diverse (ca. 81 species) and monophyletic group endemic to Australia and New Guinea (Unmack et al. 2013). They inhabit a diverse range of freshwater habitats, being found almost everywhere that freshwater is present in Australia (Allen 1989; McGuigan et al. 2000; Unmack et al. 2013). Despite their broad geographic distribution and abundance, they are nonmigratory, typically with small home ranges and strong microhabitat preferences (Hurwood and Hughes 2001; Hattori and Warburton 2003). In addition, many rainbowfish taxa have radiated relatively recently (McGuigan et al. 2000), especially the lineages endemic to Australia (Unmack et al. 2013). Given their close evolutionary relationships, strong metapopulation structures and diverse habitat preferences, these fishes represent a powerful field model for the study of local adaptation. Most species also display key attributes of a valuable laboratory model. They are small (ca. 3–9 cm), sexually dimorphic, easy to maintain and breed in captivity (they are very popular aquarium fishes), are highly fecund (between 150 and 1600 eggs) and have a short generation time, reproducing annually in the wild and at the age of 3 months in the laboratory (Pusey et al. 2001; Holdway et al. 2008).

The crimson‐spotted rainbowfish (*Melanotaenia duboulayi*) is an Australian subtropical species found in vegetated streams over a relatively broad latitudinal range in coastal drainages east of the Great Dividing Range (ca. 31.4°S to 21.4°S; Allen 1989). Temperatures along this distribution have been rising steadily, and high CO_2_ emissions projections may produce temperature as high as 10°C warmer by 2070 in the worst affected waterbodies (Meehl et al. 2007; Reisinger et al. 2014). Contemporary mean summer temperatures (ca. 21°C) are below the preferred temperature (26°C) for *M. duboulayi* tested in a thermal gradient (King and Warburton 2007). However, projected 2070 temperatures are expected to exceed the species preference and may approach levels near its critical thermal temperature (Patra et al. 2007; Beheregaray, unpublished data). Additionally, wild‐caught *M. duboulayi* exhibit substantial plastic regulatory responses to temperature stress. In a short‐term transcriptomics (RNA‐Seq) experiment, Smith et al. (2013) identified and annotated 614 upregulated and 349 downregulated genes in relation to predicted summer temperatures for 2070. These include a suite of heat‐shock genes that responded sharply to increased temperatures, and genes related to regulation of metabolic functions and developmental processes that showed mid‐range changes in expression (Smith et al. 2013). Thus, the availability of excellent candidate genes for climatic adaptation, coupled with the species’ life history attributes, makes *M. duboulayi* an ideal aquatic study system to assess molecular footprints of adaptive resilience to climate change.

In this study, we assess the adaptive potential to climate change of the subtropical Australian rainbowfish *M. duboulayi*, focusing on a genetically healthy population that is centrally located along the climatically heterogeneous latitudinal range of the species. Specifically, we use the *M. duboulayi* system to examine the effects of long‐term acclimation to increased temperature on the transcription of candidate genes which are differentially expressed in response to thermal stress, including a test for sex‐specific differences in this response. We hypothesize that there is a heritable basis underlying the variable transcriptional responses revealed during earlier experiments that used short‐term exposure to temperature treatments mimicking contemporary and future climate. Critically, we employ a reciprocal common‐garden design and incorporate information on the pedigree of experimental animals, to estimate both narrow‐sense heritability of transcription and broad‐sense genetic variation for its plasticity. Additionally, we test for functional associations with candidate genes inferred to be under temperature‐mediated selection in the wild (identified in a companion study) as a preliminary exploration of the potential adaptive value of transcriptional variation.

## Materials and methods

### Origin of broodstock, spawning & husbandry

Sixty adult *M. duboulayi* were collected using bait traps from a location in the upper reaches of the Brisbane River near the township of Fernvale (27°26′37.39″S, 152°40′12.76″E). The Brisbane River population was targeted because it is abundant compared with several *M. duboulayi* found in smaller rivers and is centrally located along the north–south range of the species in eastern Australia. In addition, a landscape genomic study of *M. duboulayi* based on over 17 000 SNPs screened in 22 populations from across the species range (Smith et al. unpublished) indicates that Brisbane River rainbowfish are part of a large representative group of populations locally adapted to climate‐related variables (hottest maximum and coldest minimum temperatures, run‐off, quarterly rainfall and annual radiation).

Fish were transported by air to the Animal House and Aquaculture facility at Flinders University in Adelaide and allowed to acclimate for 6 months. During the acclimation period, fish were maintained in single sex tanks (12 fish/100L) at 21°C under conditions of 12 h light/12 h dark. They were fed once daily on a mixture of blood worms and fish pellets. After 6 months, photoperiod was changed to a cycle of 14 h light/10 h dark to induce breeding behaviour. When fish began exhibiting nuptial coloration and gravidity, 11 mating groups of a single male and two females were selected at random and transferred to breeding tanks. Breeding tanks were 100L volume and contained ‘spawning mops’ as artificial substrate for egg deposition. Mops were removed daily in the morning and were transferred to corresponding rearing tanks. Breeding was conducted over a 4‐month period from June to October 2012. Larvae were then reared in separate, 100‐L family‐rearing tanks at 21°C under a photoperiod of 12 h light/12 h dark and fed daily, initially with rotifers and later with blood worms and fish pellets.

### Pedigree reconstruction

DNA was extracted from all parents and offspring (*n *= 199) using a modified salting‐out procedure (Sunnucks and Hales 1996). DNA quality and quantity were measured with a NanoDrop 1000 spectrophotometer. All individuals were genotyped using microsatellite markers developed for *M. australis* (Ma03, Ma09, Ma10 & Ma11; Young et al. 2009) and *M. splendida* (Ms22, Ms24 & Ms40; Zhu et al. 1998). Loci were amplified in 5 μL polymerase chain reactions (PCRs) using procedures and touch‐down cycles following (Beheregaray and Sunnucks 2000). Microsatellite fragments were scored using GENEMAPPER 4.0 (Applied Biosystems, Foster City, CA, USA), and scoring error evaluated using MICRO‐CHECKER (van Oosterhout et al. 2004). As breeding tanks contained only a single male, offspring paternity was known; maternity was inferred from resulting genotype of offspring and parents using COLONY (version 2.0.5.8; Jones and Wang 2010). Individuals were separated into full‐sib groups and kept in separate tanks until thermal treatments began.

### Thermal treatment & tissue sampling

At approximately 6 months of age, individuals from each family were assigned randomly, and in equal proportions, to one of two groups: a control representing current mean summer temperatures (21°C), and the projected mean 2070 summer temperature (31°C) for the Brisbane River region (Meehl et al. 2007; Reisinger et al. 2014). It should be noted that this projected summer temperature is below the upper thermal critical maximum (CT_MAX_ = 38°C; SD = 0.39) of *M. duboulayi* (Beheregaray, unpublished data), empirically estimated for Brisbane River rainbowfish following the method of Becker and Genoway (1979). Individuals from each temperature group were also randomly assigned to one of 19 replicate tanks. For the 31°C treatment, temperature was increased by 2°C per day until the experimental temperature was achieved. Tanks were monitored daily for signs of stress or disease associated with the higher temperature. Fish were then maintained for 80 days to approximate the typical duration of summer temperatures in the Brisbane River region.

At the end of the treatment period, fish were sacrificed using AQUI‐S^®^ solution and dissected to remove their livers for immediate RNA extraction. Although increased temperature has been shown to differentially induce transcription changes in different tissue types, we selected liver due to previous research specifically linking this tissue type to heat stress responses (Smith et al. 2013). The final data set contained 108 individuals (60 females & 48 males), assigned in approximately equal proportions to control (21°C: 26 females; 21 males) and treatment (31°C: 34 females; 27 males) groups. Nineteen families were present, 16 of which were related as paternal half‐sibs, spawned from 19 dams and 11 sires. Family size ranged from one to 16 individuals (median = 5). All families comprised of six or fewer individuals had multiple, related individuals in a corresponding half‐sib family; the three families with no half‐sib relations contained from six to 14 individuals.

### RNA extractions & qRT‐PCR

Total RNA was extracted from liver tissue using the Ambion Magmax^™^‐96 total RNA isolation kit (Thermo Fisher Scientific, Waltham, MA, USA), following Smith et al. (2013). RNA quality and concentration were measured using a Nanodrop 1000 spectrophotometer, then diluted to a standardized concentration (10 ng/μL). Each dilution was checked for DNA contamination by PCR amplification of a housekeeping gene using the SensiFAST SYBR Hi‐ROX kit (Bioline, London, UK), followed by electrophoresis on agarose gel. Samples exhibiting amplified DNA were treated with DNase MAX Kit (MO BIO) for a second time. Finally, we used random primers and the High Capacity cDNA Reverse Transcription Kit (Applied Biosystems) for cDNA synthesis, following manufacturer's protocols.

Primers were designed to amplify twelve candidate genes that are putatively differentially expressed in response to thermal stress (Smith et al. 2013) – primer sequences and related information can be found in Table S1. Most importantly, candidate genes are believed to play a role in biological processes associated with a response to thermal challenge (e.g. oxidation reduction, metabolism, regulation of transcription; see Table S2). Candidates included homologues for the following zebrafish (*Danio rerio*) genes: alanine: glyoxylate aminotransferase a (*agxta*); cytochrome P450, family 1, subfamily A (*cyp1a*); glutathione S‐transferase rho (*gstr*, a.k.a. *zgc:162356*); 3‐hydroxy‐3‐methylglutaryl‐coenzyme A synthase 1 (*hmgcs1*); heat‐shock protein 90, alpha (cytosolic), class A member 1, tandem duplicate 2 (*hsp90aa1.2*); nuclear receptor subfamily 1, group D, member 4b (*nr1d4b*); NAD(P)‐dependent steroid, dehydrogenase‐like (*nsdhl*); peroxisome proliferator‐activated receptor alpha b (*pparab*); thyrotrophic embryonic factor a (*tefa*); thioredoxin‐related transmembrane protein 2b (*tmx2b*); UDP glucuronosyltransferase 2 family, polypeptide A4 (*ugt2a4*); and ubiquinol‐cytochrome c reductase core protein IIb (*uqcrc2b*). Reference genes, to control for differences in template quantity due to variation among extractions and/or dilutions, were identified from a previous study as nondifferentially expressed between treatments (Smith et al. 2013) and included ba1 globin (*ba1*, a.k.a. *hbb*) and phosphoglycerate mutase 1a (*pgam1a*).

Quantitative PCR was performed in 96‐well plates on a StepOnePlus Real‐Time PCR System (Applied Biosystems), using a SYBR Green reporter. Reactions contained 1 μL of diluted cDNA (10 ng/μL), 5 μL of 2× SensiFAST SYBR Hi‐ROX (Bioline), 0.05 μm of each forward and reverse primer and 3.8 μL of RNase‐free water. Thermal cycles consisted of an initial denaturation step (95°C; 2 min), and 40 cycles of denaturation (95°C; 3 s), annealing (60°C; 10 s) and extension (72°C; 10 s). This was followed by a melt curve cycle of 95°C for 15 s, 60°C for 1 min and 95°C for 15 s. A single gene was amplified for a given 96‐well plate that also comprised: three negative controls; a fourfold serial dilution of a positive control, also in triplicate; three technical replicates for 30 experimental animals.

### Analyses

For each gene, raw fluorescence values of all individuals, including all technical replicates and controls, were compiled into a single data file. Initial mRNA quantities (*N*_0_) were estimated using a regression‐based procedure implemented in LinRegPCR (Ramakers et al. 2003; Ruijter et al. 2013). Replicate *N*_0_ values of each candidate gene were standardized relative to the geometric mean of *N*_0_ values for all technical replicates of reference genes for a given individual (Vandesompele et al. 2002). Standardized data were then screened for outliers: technical replicates greater than two standard deviations from their corresponding family‐by‐treatment mean were flagged and removed – on average, outlier removal resulted in the loss of a single data point (i.e. individual) per gene in the final data set (range 0–3). Following standardization and outlier removal, technical replicates were averaged by individual.

To evaluate effects of temperature and/or sex on candidate gene transcription, and to estimate additive genetic variance (*V*_A_) for transcription, we analysed data under a pedigree‐weighted, mixed effects model (i.e. the animal model) implemented in the R package ‘MCMCglmm’ (Hadfield 2010). In order to evaluate the significance of additive genetic variance for transcription, we ran two nested models: a minimal/null model including only random variation among rearing tanks, and a second model which also included ‘animal’ variance (*V*_A_); both models included coefficients describing fixed effects of temperature treatment, sex and their interaction. To ensure convergence and sufficient mixing, models were run for 25 000 000 iterations comprising an initial burn‐in of 20 000 000 iterations and a sampling chain of 5 000 000 iterations. To reduce autocorrelation of estimates, each 5000th point of the Markov chain was sampled, yielding a total of 1000 posterior estimates of model parameters. Model (i.e. *V*_A_) significance was evaluated based on the deviance information criterion (DIC); significance of fixed effects was determined by profiling the posterior distributions of estimated coefficients.

As mRNA extraction necessitated lethal sampling, individuals could not be sampled in multiple environments, thereby precluding the possibility of estimating additive genetic variance for transcriptional plasticity. However, to evaluate the potential for genetic variance in plasticity (V_G × E_), we ran two additional nested models: a model including broad‐sense genetic variance in mean transcription (i.e. variation among families in the intercept coefficient; V_G_), and a model including broad‐sense (i.e. among family) variation in the temperature (i.e. slope) coefficient. Both models also included random variation among rearing tanks and were fully parameterized for all fixed effects. Models were run with a burn‐in of 500 000 iterations, followed by an additional 500 000 iterations from which each 500th point was sampled. Significance of V_G × E_ was determined by contrasting the DIC score against both the V_G_ and previous ‘null’ models.

#### Inferring functional/adaptive value

As a tentative first inference into the potential adaptive value of variation in the candidate genes, we explored their functional relationships with a subset of genes previously identified as putatively under selection in a natural thermal gradient. In a companion paper, Smith et al. (unpublished) explored genomewide SNP variation along the latitudinal range of *M. duboulayi* and observed 1141 loci putatively under climate‐mediated selection, as identified via outlier analysis using Bayescan (Foll and Gaggiotti 2008). We first conducted a distant homology BLAST scan (gap opening and extension penalties 5 and 2; match/mismatch scores of 2 and −3; minimum percentage sequence identity 75) of the 93‐bp sequences of these outliers against the zebrafish genome (GRCz10; accessed July 28, 2015), reducing the data set to 411 annotated genes. We then used the BioMart package to retrieve identifiers (e.g. ENTREZ/NCBI accessions, official gene symbols) for use in subsequent analyses (Durinck et al. 2005).

We queried the KEGG database to identify all known molecular pathways in zebrafish comprising a co‐occurrence of at least one of the 12 candidate genes assayed for transcription and one of the annotated outlier genes (Kanehisa and Goto 2000; Kanehisa et al. 2014). Additionally, we performed a functional enrichment analysis and clustering of the two gene lists based on biological process and molecular function ontologies using DAVID (Huang et al. 2007; Sherman et al. 2007). Finally, we identified potential protein–protein interactions between members of each gene set by querying the STRING database (v10; accessed August 3, 2015; Snel et al. 2000; Szklarczyk et al. 2015). For each of the 12 candidate genes, we identified all known and predicted protein–protein interactions with the ‘highest’ confidence score (0.900). From this list, we identified 23 from the 411 genes putatively under selection and plotted their interaction network with the 12 candidate genes.

## Results

### Effects of temperature and sex on transcription

**Figure 1:**
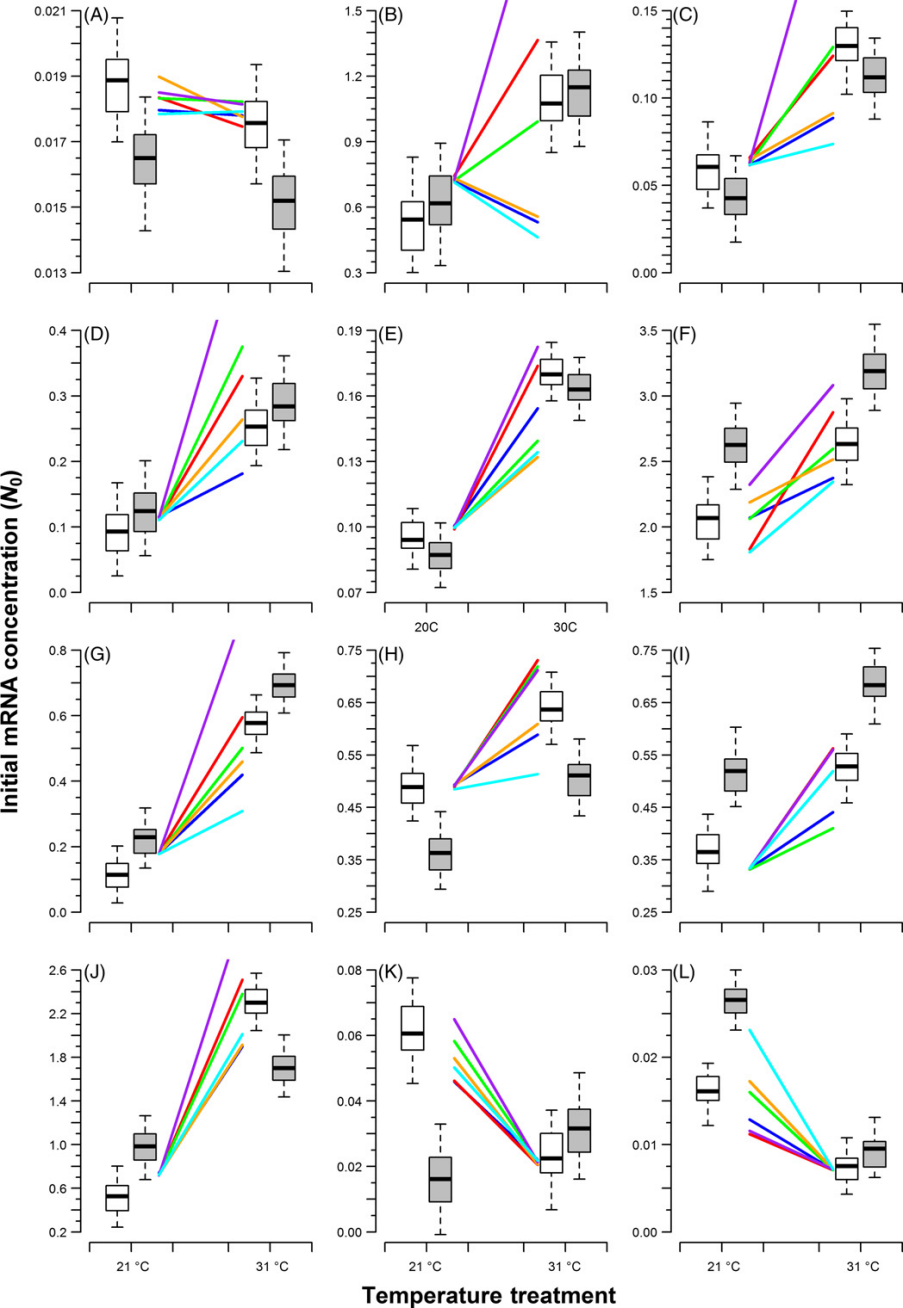
Mean initial RNA concentrations (*N*_0_), conditioned on additive genetic variance and random variation due to tank effects, as a function of sex and temperature treatment in the rainbowfish *Melanotaenia duboulayi*. Females are plotted in white and males in grey. Whiskers denote 95% PDIs; boxes indicate the quartile range of posterior estimates. (A) *tmx2b*; (B) *hmgcs1*; (C) *nsdhl*; (D) *pparab*; (E) *uqcrc2b*; (F) *agxta*; (G) *gstr*; (H) *tefa*; (I) *ugt2a4*; (J) *cyp1a*; (K) *hsp90aa1.2*; (L) *nr1d4b*. Coloured lines denote family‐specific reaction norms for 6 families represented in each temperature treatment. Note that for clarity, only female‐specific reaction norms are presented; plots of random intercept and slopes for both males and females can be found as online supplementary material (Figs S1 and S2).

With the exception of *tmx2b* (Table 1; Fig. 1A), all candidate genes exhibited a significant response to increasing temperature. Nearly all genes were significantly upregulated at 31°C (Fig. 1B–J), with the exception of *hsp90aa1.2*, for which female transcription was decreased (Fig. 1K), and *nr1d4b*, which was generally downregulated in response to increasing temperature (Fig. 1L). Five genes were also differentially expressed between sexes: *agxta* (Fig. 1F), *gstr* (Fig. 1G) and *ugt2a4* (Fig. 1I) were significantly higher in males than in female; in contrast, transcription of *tmx2b* (Fig. 1A) and *tefa* (Fig. 1H) was significantly greater in females than in males. Significant interaction effects also revealed environment dependent sex differences. Transcription of *cyp1a* was significantly higher in males at 21°C; however, this pattern was reversed at 31°C, where female levels were higher (Table 1; Fig. 1J). For *hsp90aa1.2* and *nr1d4b*, sex differences were observed only at 21°C (Table 1), with significantly greater levels of transcription for females (Fig. 1K) and males (Fig. 1L), respectively.

**Table 1:** Statistical evaluation of model coefficients describing effects of temperature treatment (Treat) and differences between sexes in the rainbowfish *Melanotaenia duboulayi*. Letters in parentheses beside gene symbols correspond to the respective panels of Fig. 1. The average fold‐change in response to temperature treatment is also reported in parentheses. For genes with a significant sex‐by‐treatment interaction, results of reduced models contrasting sexes within each temperature and describing sex‐specific temperature effects are also presented. Variance estimates are based on the fully parameterized model including all fixed effects, additive genetic variance (*V*_A_) and random variance attributable to tank effects (*V*_tank_). Estimates are based on the posterior mode (Post. Mode) and are bounded by 95% posterior density intervals (PDIs)

Gene	Fixed effects	*P*‐Value	Variance estimates			
	Model term			Post. Mode	95% PDIs	
*agxta* (F)	Treat (1.24×)	0.001	*V*_A_	0.472	0.078	0.825
	Sex	0.002	*V*_tank_	0.069	0.025	0.309
	Sex × Treat	0.774	*V*_resid_	0.196	0.045	0.505
*cyp1a* (J)	Treat (3.05×)	0.001	*V*_A_	0.105	0.024	0.383
	Sex	0.014	*V*_tank_	0.084	0.032	0.295
	Sex × Treat	0.001	*V*_resid_	0.256	0.091	0.368
	Interactions					
	21°C: M vs F	0.004				
	31°C: M vs F	0.002				
	F: 21°C vs 31°C	0.001				
	M: 21°C vs 31°C	0.001				
*gstr* (G)	Treat (4.05×)	0.001	*V*_A_	0.011	0.005	0.051
	Sex	0.006	*V*_tank_	0.009	0.003	0.030
	Sex × Treat	0.502	*V*_resid_	0.029	0.010	0.045
*hmgcs1* (B)	Treat (1.92×)	0.001	*V*_A_	0.153	0.026	0.503
	Sex	0.554	*V*_tank_	0.050	0.016	0.203
	Sex × Treat	0.182	*V*_resid_	0.255	0.058	0.410
*hsp90aa1.2* (K)	Treat (n.a.)	0.001	*V*_A_	1.0E‐03	1.7E‐04	0.002
	Sex	0.001	*V*_tank_	2.7E‐04	5.7E‐05	0.001
	Sex × Treat	0.001	*V*_resid_	3.4E‐04	3.9E‐05	0.001
	Interactions					
	21°C: M vs F	0.001				
	31°C: M vs F	0.074				
	F: 21°C vs 31°C	0.001				
	M: 21°C vs 31°C	0.448				
*nr1d4b* (L)	Treat (−2.44×)	0.001	*V*_A_	1.5E‐05	4.2E‐06	6.2E‐05
	Sex	0.001	*V*_tank_	1.2E‐05	3.4E‐06	3.6E‐05
	Sex × Treat	0.008	*V*_resid_	4.1E‐05	1.5E‐05	6.4E‐05
	Interactions					
	21°C: M vs F	0.014				
	31°C: M vs F	0.192				
	F: 21°C vs 31°C	0.001				
	M: 21°C vs 31°C	0.001				
*nsdhl* (C)	Treat (2.38×)	0.001	*V*_A_	1.4E‐03	2.4E‐04	0.004
	Sex	0.134	*V*_tank_	5.4E‐04	2.0E‐04	0.002
	Sex × Treat	0.534	*V*_resid_	2.4E‐03	8.2E‐04	0.004
*pparab* (D)	Treat (2.51×)	0.001	*V*_A_	0.005	0.001	0.032
	Sex	0.390	*V*_tank_	0.005	0.002	0.018
	Sex × Treat	0.066	*V*_resid_	0.019	0.004	0.029
*tefa* (H)	Treat (1.35×)	0.001	*V*_A_	0.008	0.001	0.035
	Sex	0.001	*V*_tank_	0.005	0.001	0.018
	Sex × Treat	0.090	*V*_resid_	0.021	0.005	0.032
*tmx2b* (A)	Treat (ns)	0.186	*V*_A_	2.0E‐06	7.8E‐07	1.9E‐05
	Sex	0.010	*V*_tank_	4.4E‐06	9.8E‐07	1.4E‐05
	Sex × Treat	0.442	*V*_resid_	1.9E‐05	9.8E‐06	2.9E‐05
*ugt2a4* (I)	Treat (1.38×)	0.001	*V*_A_	0.005	0.001	0.033
	Sex	0.001	*V*_tank_	0.005	0.001	0.021
	Sex × Treat	0.130	*V*_resid_	0.024	0.008	0.035
*uqcrc2b* (E)	Treat (1.84×)	0.001	*V*_A_	2.3E‐04	6.3E‐05	0.001
	Sex	0.292	*V*_tank_	3.3E‐04	1.1E‐04	0.001
	Sex × Treat	0.732	*V*_resid_	8.8E‐04	4.2E‐04	0.001

### Heritability and G × E for transcriptional variation

DIC for models containing both random variation due to tank effects (*V*_tank_) and *V*_A_ were lower than those of corresponding ‘null’ models (Table 2), indicating significant additive genetic variance for transcription of all candidate genes. For most genes, point estimates of *V*_tank_ were an order of magnitude less than those of *V*_A_ (Table 1). With the exception of *agxta* and *hsp90aa1.2*, residual/error variance (*V*_resid_) tended to be highest. Consequently, narrow‐sense heritability for transcription was moderate, being 0.25 on average (range = 0.11–0.54; Table 2).

**Table 2:** Summary of gene effects in the rainbowfish *Melanotaenia duboulayi*. Significance of heritability estimates is evaluated by contrasting the deviance information criterion (DIC) for a fully parameterized model (*V*_A_) with a simpler model (null) containing only random variance due to tank effects. Estimates of narrow‐sense heritability (*h* ^2^) for candidate gene expression are bounded by 95% posterior density interval estimates (PDIs). Genes whose transcription was significantly affected by rearing temperature (Temp.) are indicated by T; note that for three genes, marked *, temperature effects were sex specific. Genes with significant heritable variation in expression and those with significant family‐specific variation in temperature reaction norms (G × E) are also marked T. Adaptive and/or functional significance was inferred on the basis of candidate gene co‐occurrence with outlier genes in an annotated molecular pathway (Path.), a functional enrichment category (Enrich.), or in a predicted protein–protein interaction network (Prot.)

Gene	Model DIC		Heritability (*h* ^2^)			Statistical significance			Adaptive inference		
	Null	*V*_A_	Est.	95% PDIs		Temp.	*h* ^2^	G × E	Path.	Enrich.	Prot.
*agxta*	252.08	193.65	0.540	0.137	0.829	T	T	–	T	T	T
*cyp1a*	199.01	179.38	0.246	0.054	0.599	*	T	T	T	T	T
*gstr*	−21.84	−46.00	0.178	0.068	0.651	T	T	T	T	T	T
*hmgcs1*	223.59	182.54	0.205	0.083	0.767	T	T	T	T	–	T
*hsp90aa1.2*	−399.63	−493.37	0.518	0.197	0.876	*	T	–	T	T	T
*nr1d4b*	−724.48	−754.21	0.169	0.056	0.647	*	T	T	–	T	–
*nsdhl*	−287.79	−314.24	0.281	0.063	0.716	T	T	T	–	–	–
*pparab*	−71.46	−94.00	0.112	0.043	0.683	T	T	T	T	T	T
*tefa*	−55.05	−91.82	0.322	0.046	0.720	T	T	T	–	T	–
*tmx2b*	−815.84	−827.23	0.106	0.029	0.516	–	T	–	–	–	–
*ugt2a4*	−53.16	−79.46	0.157	0.044	0.654	T	T	T	–	–	T
*uqcrc2b*	−420.25	−428.61	0.153	0.040	0.512	T	T	T	–	T	–

Most candidate genes also exhibited evidence of significant broad‐sense genetic variation for transcriptional plasticity (Fig. S1), based on DIC comparisons with ‘null’ and V_G_ models (Tables 2 & S3). *V*_G × E_ was deemed nonsignificant for only *agxta* and *tmx2b* (Fig. S2) and was questionable for *hsp90aa1.2* given the relatively minor difference in DIC scores among models – point estimates of random slope variance (*V*_βTreat|Fam_) for these genes were also substantially lower than those of *V*_resid_ (Table S3). Of the genes with putatively significant *V*_G × E_, family‐specific reaction norms were largely in the same direction: none exhibited a crossing pattern indicative of both up‐ and downregulation in response to temperature (Fig. S1). Although based strictly on mean estimates *hmgcs1* showed a trend in this direction for three families (Fig. 1B), 95%PDIs clearly overlapped, indicating no significant plasticity in transcription associated with temperature (i.e. slope estimates not exclusive of zero, Fig. S1E). This was the pattern observed for the majority of genes: one to few families exhibited no effect of thermal treatment, whereas others significantly altered transcription in response to the temperature increase. In three genes (*cyp1a*,* gstr* & *uqcrc2b*), all families exhibited significant upregulation in response to increased temperature, although the magnitude of this effect differed.

### Functional annotations

We identified ten molecular pathways in which candidate genes (6 of 12) were found to co‐occur with genes putatively under selection (Table 3) in the natural temperature gradient of Smith et al. (unpublished). These were largely dominated by metabolic processes, but also included generation of an innate immune response (NOD‐like signalling pathway), and oocyte maturation. Enrichment analyses of overlapping GO categories revealed 28 functional clusters, with 193 of the 411 selected genes found to cluster with 8 candidate genes. Seven functional clusters comprised individual ontologies/categories with a mean fold enrichment of at least one (Table 4). Genes for transcriptional regulation and nucleotide binding were enriched at significantly higher rates than other categories, which included clusters associated with ion homeostasis and proteolysis.

**Table 3:** Molecular pathways comprising at least one candidate gene assayed for transcriptional variation in the rainbowfish *Melanotaenia duboulayi*, and genes putatively under selection in a natural temperature gradient (Selected Partners; Smith et al. unpublished). Accession numbers for the Kyoto Encyclopedia of Genes and Genomes (KEGG) database are provided for pathways associated with zebrafish homologues

KEGG ID	Pathway	Candidates	Selected partners
dre00140	Steroid hormone biosynthesis	*cyp1a*	*cyp11a1*
dre00250	Alanine, aspartate and glutamate metabolism	*agxta*	*aldh4a1*
dre00260	Glycine, serine and threonine metabolism	*agxta*	*alas2*
dre00280	Valine, leucine and isoleucine degradation	*hmgcs1*	*acaa1*
dre00480	Glutathione metabolism	*gstr*	*mgst3, ggctb*
dre00980	Metabolism of xenobiotics by cytochrome P450	*cyp1a, gstr*	*mgst3*
dre03320	PPAR signalling pathway	*pparab*	*acaa1*
dre04621	NOD‐like receptor signalling pathway	*hsp90aa1.2*	*erbb2ip*
dre04914	Progesterone‐mediated oocyte maturation	*hsp90aa1.2*	*map2k1, rps6ka3b*
dre04920	Adipocytokine signalling pathway	*pparab*	*ptpn11a*

**Table 4:** Functionally enriched clusters based on overlapping gene ontology (GO) annotations of biological process and molecular function for candidate genes and genes identified as putatively under selection in a natural temperature gradient. Overall enrichment score (Enrich.), total number of candidate and selected genes with overlapping annotations (Genes), and the number of gene ontology biological processes and molecular functions comprising the cluster (No. GO) are presented. Fold Enrichment describes the average enrichment score for all GO terms comprising the cluster

Functional cluster	Enrich.	Candidates present	Genes	No. GO	Fold enrichment
Transcriptional regulation	2.27	*nr1d4b, pparab, tefa*	45	4	1.57 (1.49–1.65)
Nucleotide binding	1.81	*hsp90aa1.2*	56	9	1.41 (1.26–1.52)
Nucleotide metabolism	0.92	*gstr*	8	33	3.27 (1.64–9.65)
Vitamin & cofactor binding	0.62	*agxta*	6	4	2.31 (1.11–3.19)
Cellular ion homeostasis	0.47	*gstr*	3	8	2.52 (1.76–3.40)
Metal ion binding	0.40	*cyp1a, gstr, pparab, uqcrc2b*	61	4	1.05 (1.01–1.12)
Peptidase proteolysis	0.18	*uqcrc2b*	13	3	0.99 (0.87–1.08)

**Figure 2:**
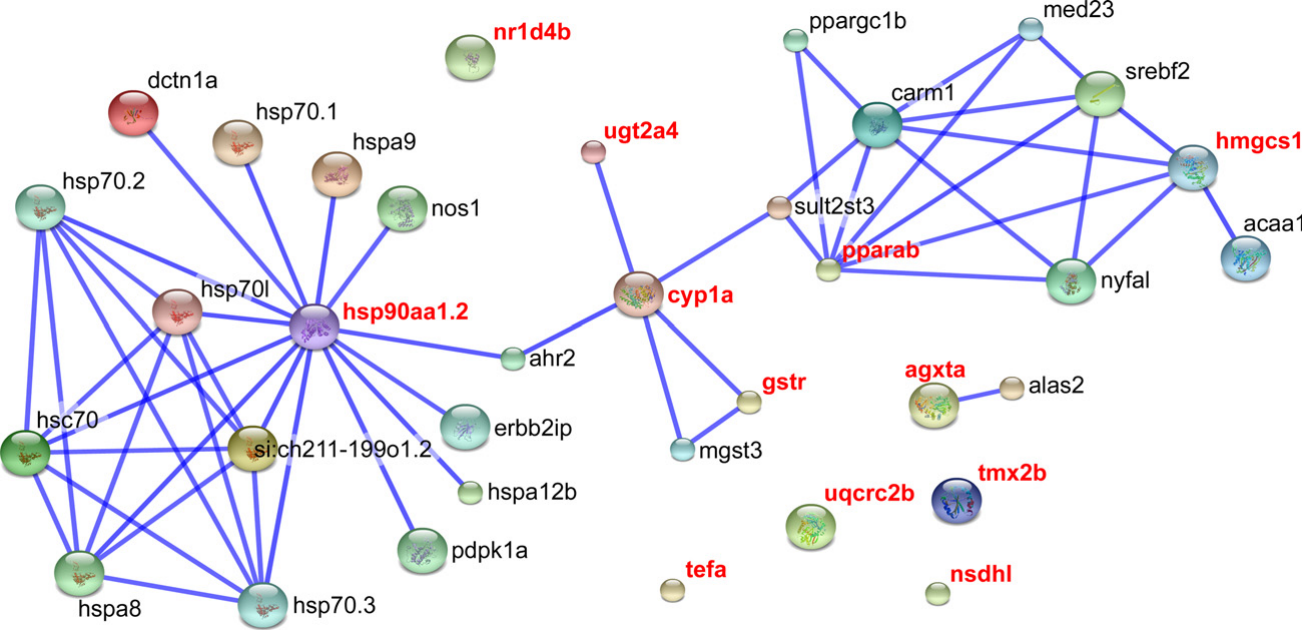
Protein–protein interaction network involving candidate genes assayed for transcriptional variation in the rainbowfish *Melanotaenia duboulayi* (bold red text), and genes putatively under selection in a natural temperature gradient (Smith et al. unpublished). Note that detailed descriptions of network edges for the two major subnetworks (hsp90aa1.2 & pparab/hmgcs1), indicating the types of interactions can be found as online supplementary material (Figs S3 and S4).

Although six candidate genes mapped to a direct protein–protein interaction with at least one putatively selected gene, two major subnetworks were most prevalent: one in which hsp90aa1.2 appeared to occupy a central node, another implicating both hmgcs1 and pparab (Fig. 2). The hsp90aa1.2 subnetwork contained the most informative functional annotations (Fig. S3), with the majority of catalysis links implicated in the deacetylation of heat‐shock factor 1 (*hsf1*) by sirtuin 1 (sirt1), and biochemical reactions associated with *hsf1* acetylation – in Fig. S3, these links are found mediated via the other heat‐shock proteins and cognates (e.g. hsc70). The remaining catalysis reaction of this subnetwork, mediated via dynactin 1a (dctn1a), mapped to the phosphorylation of polo‐like kinase 1 (*plk1*) by aurora kinase A (aurka). Annotations for the hmgcs1/pparab subnetwork were dominated by links of expression activation/induction (Fig. S4).

## Discussion

Clarifying the roles of phenotypic plasticity (environmentally induced response) and microevolution (genetic response) in scenarios of environmental change is a major research priority (Hoffmann and Willi 2008; Merilä and Hendry 2014). Whilst under certain conditions plasticity can be adaptive (Gotthard and Nylin 1995; Ghalambor et al. 2007), genetic responses are generally a requisite for long‐term persistence to predicted climate changes (Bradshaw and Holzapfel 2006). Moreover, the potential for an evolved response requires demonstration of heritable variation in traits affecting individual performance under new environmental conditions (Gienapp et al. 2008; Charmantier and Gienapp 2014). We can draw two conclusions from our long‐term common‐garden experiment of functional responses to predicted climate change with the Australian rainbowfish *M. duboulayi*. The first is that increases in seasonal austral summer temperature projected under a high emissions scenario are likely to have a significant effect on the transcriptional profiles of this subtropical rainbowfish. The second is that even in the face of significant temperature effects, there is a variation in both transcription and the transcriptional response to increased temperatures. Most importantly, this variation has an underlying genetic basis and, as such, might allow for an evolved response.

### Additive genetic variance and adaptive potential

Perhaps more important than confirmation of the transcriptional effects of projected temperature increases, this study adds to the growing body of evidence that transcriptional variance has an underlying heritable basis. Much of the previous work in the field has been dominated by studies designed to identify quantitative trait loci responsible for transcriptional variation (eQTL, Gibson and Weir 2005; Skelly et al. 2009). Whilst this line of enquiry has provided many valuable insights into the regulatory nature of gene expression, classical pedigree‐based analyses of variance components may provide more reliable estimates of the fraction of transcriptional variance due to additive genetic effects (discussed in Leder et al. 2015). Thus, not only is this a highly cost‐effective approach to understanding the genetics of transcriptional variance, but also it has the potential to yield more accurate estimates of the parameters required to predict a response to changing selection pressures. Yet to date surprisingly few studies investigating the transcriptional effects of changing environmental stressors have taken such a pedigree‐based approach (though see Côté et al. 2007; Roberge et al. 2007; Debes et al. 2012).

Although this study does provide requisite proof of genetic variation, at present we do not know how transcriptional variance in these candidate genes might affect fitness. In fact, the general importance of transcriptional variance as a contributor to fitness remains a point of contention (Feder and Walser 2005; Evans 2015). Given the difficulty of quantifying fitness, it is perhaps unsurprising that few studies have attempted to integrate fitness metrics into the analysis of transcriptional variation in wild vertebrate models (but see McCairns and Bernatchez 2010). Nevertheless, we may infer that some contributions to fitness must be conferred given the signatures of selection observed in transcriptional variation in natural populations. For example, stickleback populations inhabiting a broad latitudinal gradient in northern Europe show signatures of directional selection in nearly 16% of all transcripts, with many enriched for functional groups associated with temperature stress (Leder et al. 2015). Likewise, 22% of transcripts assayed in killifish adapted to a steep temperature gradient along the coast of eastern North America also show evidence of selection (Whitehead and Crawford 2006). These studies demonstrate that transcriptional variation can clearly respond to selection and aid in the adaptation to divergent thermal environments. However, establishing a direct link to fitness remains an important next step if we are to understand its role in *M. duboulayi*'s adaptive potential in a changing thermal environment.

### Inference from predicted functional associations

Although function alone does not imply fitness, it does contribute to whole organism performance, and so may help to inform our understanding of the targets of selection (Dalziel et al. 2009). Many of the candidate genes are known to be directly involved in pathways associated with oxidative stress (e.g. *gstr*; Hermes‐Lima and Zenteno‐Savín 2002) and the regulation of metabolism (e.g. nr1d4b; Desvergne et al. 2006), both physiological processes likely to be affected by a warming thermal environment. And whilst new analyses suggest that mRNA levels might be a far better predictor of protein abundance than previously believed (Li and Biggin 2015), increased protein production is not necessarily equivalent to increased functional activity (Feder and Walser 2005). However, in evolved/steady‐state comparisons, transcriptional variation can correspond remarkably well to differences in enzyme activity (Nikinmaa et al. 2013). Given the extensive acclimation period of this study, we are likely safe to assume that current transcriptional data are reflective of steady‐state levels typical of summer conditions, both current and projected. As such, it is plausible that a degree of functional significance is captured in these data and that the most likely candidates in the physiologically mediated function/fitness nexus should be found at the functional intersection with genes under selection in the wild.

### A role for plasticity?

All genes exhibited significant heritability for transcription. Although the proportion of total phenotypic variance explained by additive genetic effects was moderate (Table 2), estimates are similar to that observed in various taxa (reviewed in Leder et al. 2015). Whether these are too low to effectively respond to selection exerted by increasing temperature remains an open question. For example, in UK populations of the great tit (*Parus major*), heritability for breeding time is similarly modest (*h* ^2^ = 0.16), and insufficient to account for shifts which appear to track environmentally induced changes in an important prey item's phenology (Charmantier et al. 2008). In this example, plasticity in breeding time appears to have facilitated the shift. Given the labile nature of transcription, this raises the possibility of plasticity also facilitating a shift towards new optima beyond the limits of existing genetic variation.

Plasticity in the form of acclimation seems an unlikely long‐term strategy. Indeed, species with an evolutionary history in highly variable thermal environments (i.e. those frequently adjusting/acclimating to changing conditions) may currently exist within their tolerance limits for physiological stress (Tomanek 2010), and so may have limited plastic acclimation potential beyond this to meet additional physiological challenges imposed by climate change. The view with respect to transcriptional plasticity is also ambiguous. For example, a comparison of acute versus chronic (3 weeks) thermal stress in the bald notothen (*Pagothenia borchgrevinki*) revealed that mRNA levels of genes associated with ROS scavenging were increased in acute exposure but did not differ between control and chronic exposure; most critically, chronically exposed fish showed significantly higher levels of oxidative damage (Almroth et al. 2015). It should be noted, however, that this observation comes from a highly stenothermal species, a group which may lack a typical heat‐shock response (Tomanek 2010). In contrast, brown trout (*Salmo trutta*) sampled from different thermal environments exhibit patterns of G × E for transcription suggestive of either a role for plasticity during adaptation to different temperatures, or the evolution of plasticity within them (Meier et al. 2014).

One possible caveat to the role of transcriptional plasticity in adaptation, however, may be seen in the yeast model. Although studied in the context of nutritional environmental quality and not temperature, significant G × E for transcription was largely restricted to genes unlikely to have unique essential functions, for example paralogous genes (Landry et al. 2006). If this observation also holds true for other taxa, this might limit the number and/or types of genes with adequate heritable plasticity. One likely candidate, however, remains the PPAR pathway. Transcriptional regulation via PPARs (e.g. *pparab*) may play a role in acclimation to both temperature and nutritional stress (Seebacher et al. 2010). In freshwater sticklebacks, plasticity in the transcription of *pparaa* (a *pparab* paralogue) is thought to have played a role in the colonization of a broader range of thermal environments than experienced by the ancestral population (Morris et al. 2014).

In all cases, for plasticity to play a meaningful role in adaptation to a changing environment, it must move the phenotype towards the new fitness peak and it should be heritable (Gotthard and Nylin 1995; Ghalambor et al. 2007). In subtropical rainbowfish, differences in temperature reaction norms indicate considerable variation in the potential response to increasing temperature (Fig. S1). Likewise, the relatively wide‐ranging posterior density intervals for certain families can be interpreted as a proxy for plasticity in the classical sense (i.e. the ability of a given genotype to produce a range of phenotypes in different environments). Moreover, we suggest that significant variation among families in the slope coefficient for nine of the twelve candidate genes is representative of broad‐sense heritability for plasticity. As such, if plasticity itself becomes an important determinant of fitness, there may be sufficient genetic variation upon which selection might act. Given that increased temperature variations are also a feature of most projections for the region (Reisinger et al. 2014), such potential for adaptive plasticity may play an important role in the persistence of *M. duboulayi* along the east coast of Australia.

### Acclimation time influences transcription pattern

For many candidate genes, the transcriptional response to a season‐length duration of increased temperature showed patterns similar to those exhibited following short‐term (i.e. 14d) exposure (Smith et al. 2013). Three in particular (*hmgcs1*,* nr1d4b* & *nsdhl*) were not only consistent between studies of *M. duboulayi*, but also with respect to observations in threespine stickleback (*Gasterosteus aculeatus*) subjected to an acute temperature stress (Leder et al. 2015). That the range of fold‐change values observed among these studies was also remarkably similar would suggest that these genes may play a general role in the thermal stress response and/or thermal acclimation in teleost fishes – indeed increased liver transcription of *hmgcs1* has also been reported in zebrafish (*Danio rerio*) exhibiting metabolic and oxidative stress (Hugo et al. 2012; Zhang et al. 2012; Tsedensodnom et al. 2013), both conditions associated with thermal stress. Similarly, two other candidate genes (*gstr* & *uqcrc2b*) were consistently upregulated in this and the previous study of *M. duboulayi*. However, seven of the twelve genes studied exhibited patterns of transcription opposite of that inferred by transfrag quantitation of RNA‐Seq data.

Given the preponderance of transcription differences between sexes, and the fact that only males were assayed in the previous RNA‐Seq study, sex‐specific patterns are one potential explanation for observed discrepancies. However, only *tmx2b* and *hsp90aa1.2* show sufficient variability (i.e. overlapping posterior density intervals; Fig. 1A and K) from which biased sampling might potentially suggest increased transcription in response to temperature, as observed previously. Moreover, although males show a trend towards increased *hsp90aa1.2* transcription, this is neither significant (Table 1) nor within an order of magnitude of the degree of fold change previously reported (ca. 140×). Conversely, threespine sticklebacks have been shown to exhibit a ca. 10‐fold increase of *hsp90aa1.2* in liver tissue immediately following a temperature increase (Leder et al. 2015). Likewise, killifish (*Austrofundulus limnaeus*) show levels of *hsp90α* induction that appear to increase over the first 24 h of thermal treatment, but maintain a relatively constant level of upregulation over a 14‐d period (Podrabsky and Somero 2004). The common element between these experiments and previous observations in *M. duboulayi* is the relatively short duration of exposure, likely occurring within a window of physiological stress/disruption of homeostasis. In contrast, current observations were made on fish likely acclimated to the increased temperature.

Acclimation time is known to affect the expression of a suite of physiological traits (Schulte et al. 2011; Healy and Schulte 2012), and molecular responses to thermal challenge are no exception. With respect to mRNA transcription, expression is dependent not only upon acclimation time, but also the degree of thermal change experienced (Currie and Schulte 2014). Whilst the common, immediate response to increased temperature involves initiation of a stress response, there appears to be considerable variability in the length of time required for homeostasis to be re‐established. For many temperate‐zone fishes, including both stenothermal and eurythermal species, transcriptome‐wide patterns of expression have been observed at steady state within a 4‐week acclimation period (Vornanen et al. 2005; Logan and Somero 2010). In contrast, transcriptional patterns consistent with those of acute thermal stress persist in zebrafish following 4 weeks of exposure to elevated temperatures (Vergauwen et al. 2010). Little is known about the duration and/or periodicity of the molecular response to thermal stress in *M. duboulayi* specifically; as such, we can only assume that transcript levels following 80 days of acclimation to experimental temperatures are at steady state. Irrespective, given the choice of experimental temperatures, these observations are likely to capture some elements of the transcriptional mechanisms underlying acclimatization to future summer seasons.

It should also be noted that previous observations were made on wild‐caught fish. Although acclimated to control temperature for 1 month prior to experimentation, it is unknown to what extent experimental individuals may have experienced different temperatures when inhabiting a naturally heterogeneous thermal environment. Temperature variation during embryonic development can have a significant impact on thermal acclimation potential and the transcriptomic response of adult fish to thermal stress (Schaefer and Ryan 2006; Scott and Johnston 2012). Thus, discrepancies could merely reflect differences between fish reared under fluctuating versus constant temperatures during a critical developmental stage. Whilst this may open reasonable debate regarding the extent to which observations on model animals reared under artificially homogeneous conditions can be extrapolated to wild populations, we would re‐iterate that this is outweighed by a significant advantage: the ability to disentangle environmental and genetic sources of variation.

In conclusion, this study provides a tangible example that can be added to the debate surrounding adaptive evolution in response to climate change. Evidence of heritability in candidate gene expression, in addition to broad‐sense genetic variation in transcriptional plasticity, speaks to the potential for an adaptive response to future increases in environmental temperature. Additionally, molecular pathways of candidate genes were found to co‐occur with genes putatively under climate‐mediated selection in wild *M. duboulayi* populations. Such findings are essential to meaningful discussions of adaptive change, but are sorely lacking in many studies of climate change adaptation. Although establishing a direct link between transcriptional variation and fitness remains essential if we are to understand its role in *M. duboulayi*'s adaptive potential in a changing thermal environment, our study represents important first steps towards that end.

## Conflict of interest

The authors declare no conflict of interest.

## Data archiving

Data available from the Dryad Digital Repository: http://dx.doi.org/10.5061/dryad.h40d8

## Supporting information


**Table S1**. Primer sequences of reference and candidate genes.
**Table S2** Putative protein family identity and functional role(s) of candidate genes in the rainbowfish *Melanotaenia duboulayi*.
**Table S3**. Variance estimates from nested mixed‐effects models in the rainbowfish *Melanotaenia duboulayi*.
**Figure S1**. Family‐specific reaction norms for genes with significant G × E for expression in the rainbowfish *Melanotaenia duboulayi*.
**Figure S2**. Family‐specific reaction norms for genes with non‐significant or marginal/questionable G × E for expression in the rainbowfish *Melanotaenia duboulayi*.
**Figure S3**. Interactions between proteins for the hsp90aa1.2 sub‐network.
**Figure S4**. Interactions between proteins for the pparab/hmgcs1 sub‐network.
