# Supplementary material for: The adaptive potential of subtropical rainbowfish in the face of climate change: heritability and heritable plasticity for the expression of candidate genes
Source: Evol Appl. 2016 Feb 18;9(4):531–45. doi: 10.1111/eva.12363 (PMC4831457; doi:10.1111/eva.12363)

Online supplementary materials

Table S1. Primer sequences of reference and candidate genes. Gene identity was determined on the basis of sequence similarity of the transcript fragment sequence used for primer design with the nearest zebrafish (*Danio rerio*) homologue – e value and percent sequence identity (Ident.) are presented, along with the NCBI accession for the mapped homologues.

Gene	NCBI	E Value	Ident.	Type	Forward Primer	Reverse Primer	Amplicon Size
ba1	30216	4.00E-39	79.2	ref	TGGGGAAATTGGACCCCAGG	GGTTGCCGAATGACCCGAAG	83
pgam1a	323107	0.00E-0	80.6	ref	GGACCGTCGCTATGCAGACT	GCAGCAATTAGCACCCGCTT	141
agxta	436603	1.00E-25	78.6	target	ACTCTGCGATGGAGTGTGCC	TTCTGTAGCCCGCTCTCCCC	95
cyp1a	140634	8.00E-50	66.1	target	GGAGCTGCTTGGCTTGGTGA	TTGCCACTGCCTGTCACCTG	60
gstr	564619	5.00E-09	62.8	target	AGGACACACCAGGGCAGGAT	TTGATGCAACGGCAGCAGGT	72
hmgcs1	394060	6.00E-49	81.7	target	GAGGTCTGCGGGGAACACAC	ACAGCTTTCCGTCCACCACG	90
hsp90aa1.2	565155	0.00E-0	84.3	target	GCCGTCCAACCATACGTGCT	GTCACTGTCTCCAACCGCCT	68
nr1d4b	571948	4.00E-126	77.6	target	CTCCTGACGTGCCGAGGTTC	TCGGGAGATGTGCCAAGGGA	79
nsdhl	550369	1.00E-47	74.3	target	CCAGTGTTGTGTTCGAGGGCA	GTCGATGGGCTTCTTGGCGT	74
pparab	557714	3.00E-92	79.1	target	AAGCCCTCTTCGCCATGCTG	CGGGTGATGAAGCCAGAGCC	79
tefa	30674	3.00E-89	77.9	target	GCTTCAGCTCCTCCTCGCTG	CTGTCGAGCGTTCCTGGTGG	70
tmx2b	415203	2.00E-107	81.9	target	AGCTTCCATCGCTGGTGCTG	CGGCTCTGCCCTTCTTGTCC	78
ugt2a4	791613	8.00E-86	72.6	target	GCCATCTACCACGGTGTTCCC	GTCTGGCTGGTCCGCAAACA	57
uqcrc2b	322549	1.00E-164	69.5	target	TGCTGACTGGCACACCACAG	GTGAGATCCGTCTGCCCAGC	62

Table S2. Putative protein family identity and functional role(s) of candidate genes in the rainbowfish *Melanotaenia duboulayi*. Biological process (BP) and molecular function (MF) categories and accessions are provided for Gene Ontology (GO) annotations. KEGG annotations and accessions are also provided for molecular pathways in which zebrafish homologues are members.

Gene	Putative Annotation			
	Protein Family	Ontology	Accession	Description
agxta	serine pyruvate aminotransferase	MF	GO:0008483	transaminase activity
	alanine glyoxylate aminotransferase		GO:0019842	vitamin binding
	agt		GO:0030170	pyridoxal phosphate binding
			GO:0048037	cofactor binding
		KEGG	dre00250	alanine, aspartate and glutamate metabolism
			dre00260	glycine, serine and threonine metabolism
			dre00630	glyoxylate and dicarboxylate metabolism
			dre01200	carbon metabolism
			dre04146	peroxisome
cyp1a	cytochrome p450	BP	GO:0009410	response to xenobiotic stimulus
			GO:0055114	oxidation reduction
		MF	GO:0005506	iron ion binding
			GO:0009055	electron carrier activity
			GO:0016712	oxidoreductase activity
			GO:0020037	heme binding
			GO:0046906	tetrapyrrole binding
		KEGG	dre00140	steroid hormone biosynthesis
			dre00380	tryptophan metabolism
			dre00830	retinol metabolism
			dre00980	metabolism of xenobiotics by cytochrome P450
gstr	glutathione s transferase	BP	GO:0006826	iron ion transport
zgc:162356	rho-class glutathione s transferase		GO:0006879	cellular iron ion homeostasis
	gst class theta		GO:0009617	response to bacterium
			GO:0055082	cellular chemical homeostasis
		MF	GO:0005506	iron ion binding
		KEGG	dre00480	glutathione metabolism
			dre00980	metabolism of xenobiotics by cytochrome P450
hmgcs1	hydroxymethylglutaryl coa synthase	BP	GO:0006720	isoprenoid metabolic process
	hmg coa synthase		GO:0008299	isoprenoid biosynthetic process
	3 methylglutaryl coenzyme a synthase		GO:0008610	lipid biosynthetic process
		MF	GO:0004421	hydroxymethylglutaryl-CoA synthase activity
			GO:0046912	transferase activity (acyl groups converted into alkyl on transfer)
		KEGG	dre00072	synthesis and degradation of ketone bodies
			dre00280	valine, leucine and isoleucine degradation
			dre00650	butanoate metabolism
			dre00900	terpenoid backbone biosynthesis

Table S2. *Continued*.

Gene	Putative Annotation			
	Protein Family	Ontology	Accession	Description
hsp90aa1.2	heat shock	BP	GO:0006457	protein folding
		MF	GO:0000166	nucleotide binding
			GO:0001882	nucleoside binding
			GO:0005524	ATP binding
			GO:0032553	ribonucleotide binding
			GO:0051082	unfolded protein binding
		KEGG	dre04141	protein processing in endoplasmic reticulum
			dre04621	NOD-like receptor signaling pathway
			dre04914	progesterone-mediated oocyte maturation
nr1d4b	nuclear receptor subfamily 1, group d member	BP	GO:0006355	regulation of transcription, DNA-dependent
			GO:0051252	regulation of RNA metabolic process
		MF	GO:0003677	DNA binding
			GO:0003700	transcription factor activity
			GO:0003707	steroid hormone receptor activity
			GO:0004879	ligand-dependent nuclear receptor activity
			GO:0004887	thyroid hormone receptor activity
			GO:0030528	transcription regulator activity
nsdhl	sterol 4 alpha carboxylate 3 dehydrogenase, decarboxylating	BP	GO:0006694	steroid biosynthetic process
			GO:0008202	steroid metabolic process
			GO:0008610	lipid biosynthetic process
			GO:0055114	oxidation reduction
		MF	GO:0003854	3-beta-hydroxy-delta5-steroid dehydrogenase activity
			GO:0033764	steroid dehydrogenase activity (CH-OH group donors)
		KEGG	dre00100	steroid biosynthesis
pparab	peroxisome proliferator activated receptor	BP	GO:0006355	regulation of transcription, DNA-dependent
	ppar nuclear receptor subfamily 1		GO:0009266	response to temperature stimulus
			GO:0051252	regulation of RNA metabolic process
		MF	GO:0003700	transcription factor activity
			GO:0003707	steroid hormone receptor activity
			GO:0004879	ligand-dependent nuclear receptor activity
			GO:0008270	zinc ion binding
			GO:0030528	transcription regulator activity
			GO:0043565	sequence-specific DNA binding
		KEGG	dre03320	PPAR signaling pathway
			dre04920	adipocytokine signaling pathway

Table S2. *Continued*.

Gene	Putative Annotation			
	Protein Family	Ontology	Accession	Description
tefa	thyrotroph embryonic factor	BP	GO:0006355	regulation of transcription, DNA-dependent
			GO:0051252	regulation of RNA metabolic process
		MF	GO:0003700	transcription factor activity
			GO:0030528	transcription regulator activity
			GO:0043565	sequence-specific DNA binding
			GO:0046983	protein dimerization activity
tmx2b	thioredoxin related transmembrane 2 precursor	BP	GO:0019725	cellular homeostasis
			GO:0045454	cell redox homeostasis
ugt2a4	udp glucuronosyltransferase precursor	BP	GO:0009813	flavonoid biosynthetic process
	udpgt		GO:0052696	flavonoid glucuronidation
			GO:0032870	cellular response to hormone stimulus
		MF	GO:0016757	transferase activity, transferring glycosyl groups
			GO:0016758	transferase activity, transferring hexosyl groups
uqcrc2b	cytochrome b c1 complex subunit 2, mitochondrial precursor	BP	GO:0006508	proteolysis
	ubiquinol cytochrome c reductase complex core 2	MF	GO:0004222	metalloendopeptidase activity
			GO:0008237	metallopeptidase activity
			GO:0008270	zinc ion binding
			GO:0070011	peptidase activity, acting on L-amino acid peptides
		KEGG	dre00190	oxidative phosphorylation
			dre04260	cardiac muscle contraction

Table S3. Variance estimates from nested mixed-effects models in the rainbowfish *Melanotaenia duboulayi*. All models include the fixed effects of temperature treatment, sex and their interaction term (estimates not shown). The null model includes only random variance associated with tank effects (**V_{tank}**) and residual/error variance (**V_{resid}**). A model of broad-sense genetic variance (V_G) also includes among-family variance in the intercept coefficient (**$V_{\beta0|Fam}$**); is next; a model incorporating broad-sense genetic variation for plasticity (V_{GxE}) also includes among-family variance in the slope coefficient (**$V_{\beta Treat|Fam}$**). Estimates are based on the posterior mode (**Est.**), and are bounded by 95% posterior density intervals (**95% PDIs**). Model evaluation is by the deviance information criterion (**DIC**), with the favoured model highlighted in bold text.

**Gene**	**Model**	**DIC**	**V_{tank}**			**$V_{\beta0\|Fam}$**			**$V_{\beta Treat\|Fam}$**			**V_{resid}**		
			**Est.**	**95% PDIs**		**Est.**	**95% PDIs**		**Est.**	**95% PDIs**		**Est.**	**95% PDIs**	
agxta	null	252.08	0.206	4.2E-02	4.3E-01							0.551	0.394	0.711
	V_G	**241.09**	0.006	4.8E-17	2.6E-02	0.298	6.8E-02	6.8E-01				0.490	0.355	0.620
	V_{GxE}	245.84	0.130	9.2E-17	3.7E-01	0.193	1.7E-16	6.7E-01	0.192	1.0E-16	6.3E-01	0.486	0.334	0.699
cyp1a	null	199.01	0.124	4.4E-15	2.7E-01							0.340	0.240	0.466
	V_G	198.89	0.120	9.4E-16	2.6E-01	0.004	6.9E-17	1.8E-02				0.342	0.250	0.472
	V_{GxE}	**186.44**	0.010	1.3E-16	6.6E-02	0.003	7.8E-17	1.8E-02	0.254	7.0E-17	5.3E-01	0.298	0.216	0.392
gstr	null	-21.84	0.013	5.3E-06	2.8E-02							0.042	0.031	0.057
	V_G	-19.33	0.009	1.6E-16	2.7E-02	0.004	1.2E-16	1.9E-02				0.042	0.030	0.055
	V_{GxE}	**-58.41**	1.6E-04	8.6E-17	9.3E-04	6.7E-05	9.8E-17	3.3E-04	0.057	1.6E-02	1.1E-01	0.029	0.021	0.038
hmgcs1	null	223.59	0.092	1.7E-15	2.3E-01							0.419	0.282	0.560
	V_G	217.90	0.032	1.0E-16	1.5E-01	0.097	1.9E-16	2.6E-01				0.392	0.267	0.511
	V_{GxE}	**195.02**	0.003	1.1E-16	1.6E-02	0.004	8.4E-17	2.5E-02	0.349	6.5E-02	7.2E-01	0.312	0.217	0.406
hsp90aa1.2	null	-399.63	3.4E-05	1.1E-16	2.5E-04							1.3E-03	9.6E-04	0.002
	V_G	-402.63	4.2E-05	9.1E-17	2.5E-04	1.8E-04	8.0E-17	8.9E-04				1.2E-03	8.0E-04	0.002
	V_{GxE}	**-403.44**	4.4E-05	8.2E-17	3.1E-04	3.0E-04	7.6E-17	1.3E-03	3.9E-05	7.1E-17	2.7E-04	1.2E-03	7.6E-04	0.002
nr1d4b	null	-724.48	4.8E-06	7.8E-17	2.0E-05							6.3E-05	4.3E-05	8.1E-05
	V_G	-725.73	4.3E-06	1.3E-16	2.2E-05	3.3E-06	1.1E-16	2.0E-05				6.1E-05	4.1E-05	8.0E-05
	V_{GxE}	**-743.78**	4.6E-07	8.2E-17	2.2E-06	4.8E-05	1.1E-16	1.0E-04	1.9E-07	1.2E-16	1.0E-06	4.9E-05	3.5E-05	6.5E-05

Table S3. *Continued*.

Gene	Model	DIC	V_{tank}			$V_{\beta 0\|Fam}$			$V_{\beta Treat\|Fam}$			V_{resid}		
			Est.	95% PDIs		Est.	95% PDIs		Est.	95% PDIs		Est.	95% PDIs	
nsdhl	null	-287.79	9.6E-04	1.5E-09	2.0E-03							0.004	0.003	0.005
	V_G	-287.56	3.9E-04	7.1E-17	1.6E-03	5.8E-04	1.1E-16	1.8E-03				0.004	0.002	0.005
	V_{GxE}	**-317.67**	4.4E-05	8.9E-17	3.0E-04	5.0E-05	1.1E-16	3.4E-04	0.003	7.7E-04	5.9E-03	0.003	0.002	0.003
pparab	null	-71.46	0.009	1.4E-03	2.0E-02							0.026	0.019	0.034
	V_G	-69.79	0.007	2.0E-16	1.6E-02	0.002	1.0E-16	1.1E-02				0.026	0.019	0.035
	V_{GxE}	**-80.38**	0.001	1.2E-16	6.7E-03	4.0E-04	1.4E-16	3.3E-03	0.019	3.3E-13	4.1E-02	0.023	0.017	0.030
tefa	null	-55.05	0.003	1.1E-16	1.2E-02							0.032	0.022	0.042
	V_G	-55.96	0.002	1.3E-16	8.4E-03	0.002	1.5E-16	1.2E-02				0.032	0.022	0.043
	V_{GxE}	**-71.93**	3.7E-04	4.9E-17	2.3E-03	3.7E-04	7.6E-17	2.4E-03	0.026	1.7E-15	6.6E-02	0.026	0.018	0.035
tmx2b	null	**-815.84**	5.7E-06	1.0E-15	1.4E-05							2.4E-05	1.6E-05	3.1E-05
	V_G	-813.87	4.7E-06	1.3E-16	1.3E-05	3.8E-07	1.0E-16	2.6E-06				2.4E-05	1.7E-05	3.3E-05
	V_{GxE}	-813.69	4.2E-06	1.5E-16	1.3E-05	1.4E-06	8.2E-17	8.8E-06	1.3E-06	9.3E-17	8.2E-06	2.4E-05	1.6E-05	3.2E-05
ugt2a4	null	-53.16	0.002	1.0E-16	1.1E-02							0.034	0.024	0.045
	V_G	-53.51	0.002	9.8E-17	1.2E-02	9.7E-04	1.2E-16	6.7E-03				0.033	0.023	0.043
	V_{GxE}	**-65.73**	0.001	7.6E-17	3.7E-03	1.9E-04	7.1E-17	9.7E-04	0.018	2.1E-16	4.7E-02	0.028	0.019	0.039
uqcrc2b	null	-420.25	5.1E-04	6.9E-05	1.1E-03							1.0E-03	7.2E-04	1.3E-03
	V_G	-419.41	5.1E-04	2.6E-16	1.1E-03	1.2E-05	9.4E-17	5.5E-05				1.0E-03	7.3E-04	1.3E-03
	V_{GxE}	**-429.42**	3.3E-05	7.0E-17	1.9E-04	9.1E-06	1.1E-16	5.2E-05	8.9E-04	2.2E-16	1.9E-03	9.1E-04	6.6E-04	1.2E-03

Figure S1. Family-specific reaction norms for genes with significant G×E for expression in the rainbowfish *Melanotaenia duboulayi*. Mean initial RNA concentrations (N_0) are conditioned on random variation due to tank effects and among family variation in intercept and slope coefficients. Females are plotted left, males right. Each of 6 families are denoted by a unique colour, with families represented in each temperature treatment. Whiskers denote 95% PDIs; boxes indicate the quartile range of posterior estimates. Shaded regions denote the 95% range of posterior estimates conditioned on additive genetic effects only (as per Figure 1).

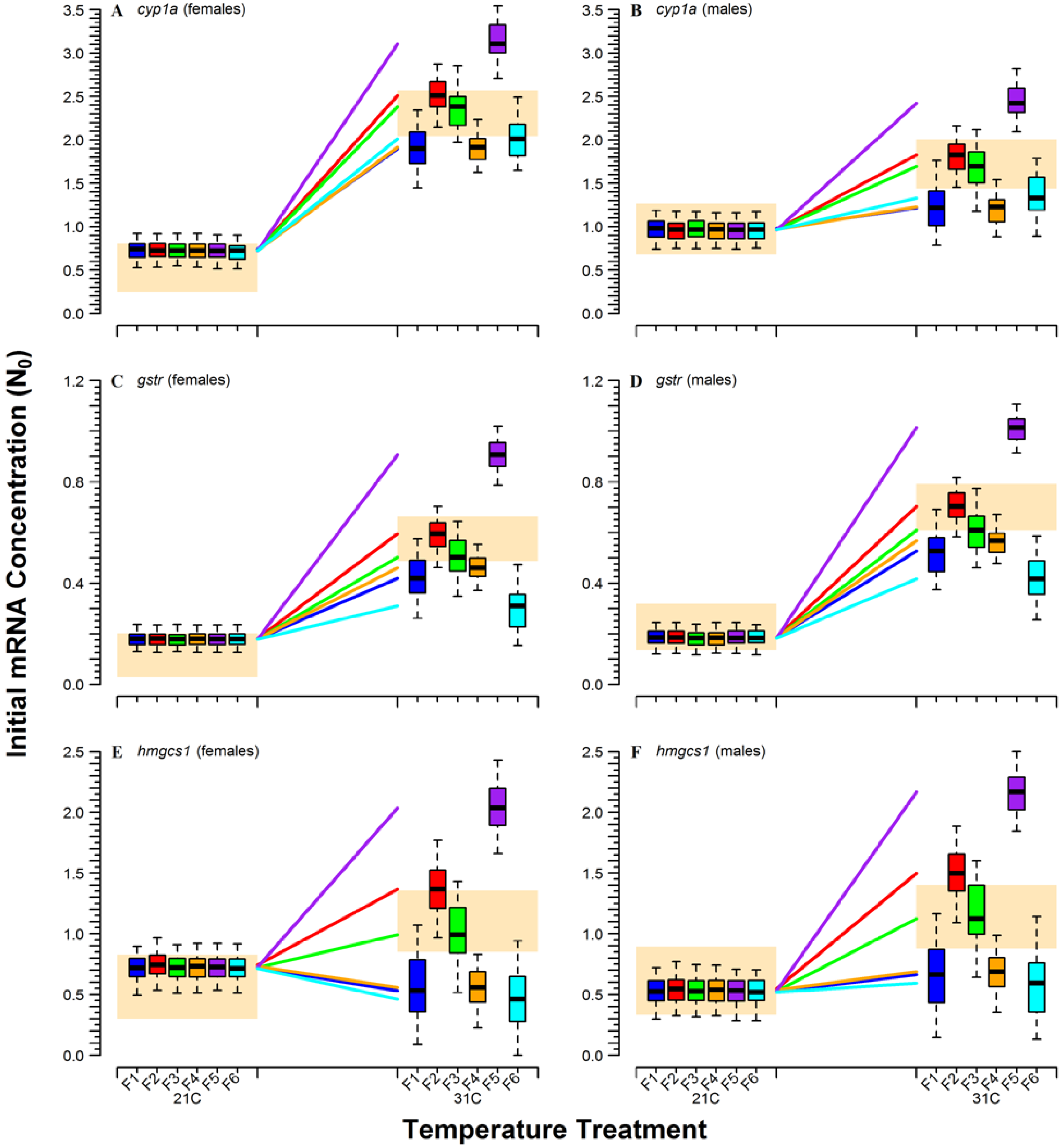

Figure S1. *Continued*

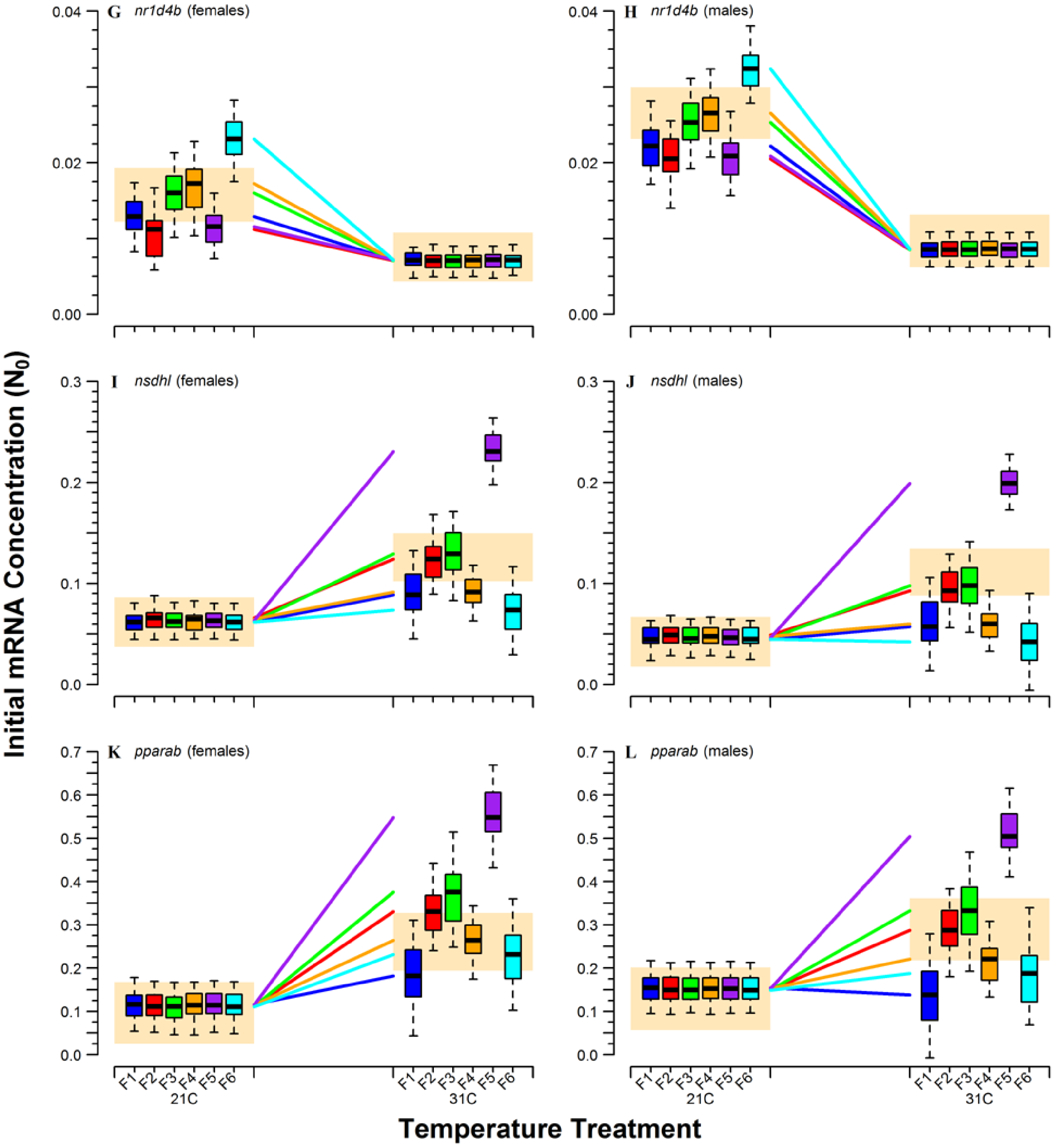

Figure S1. *Continued*

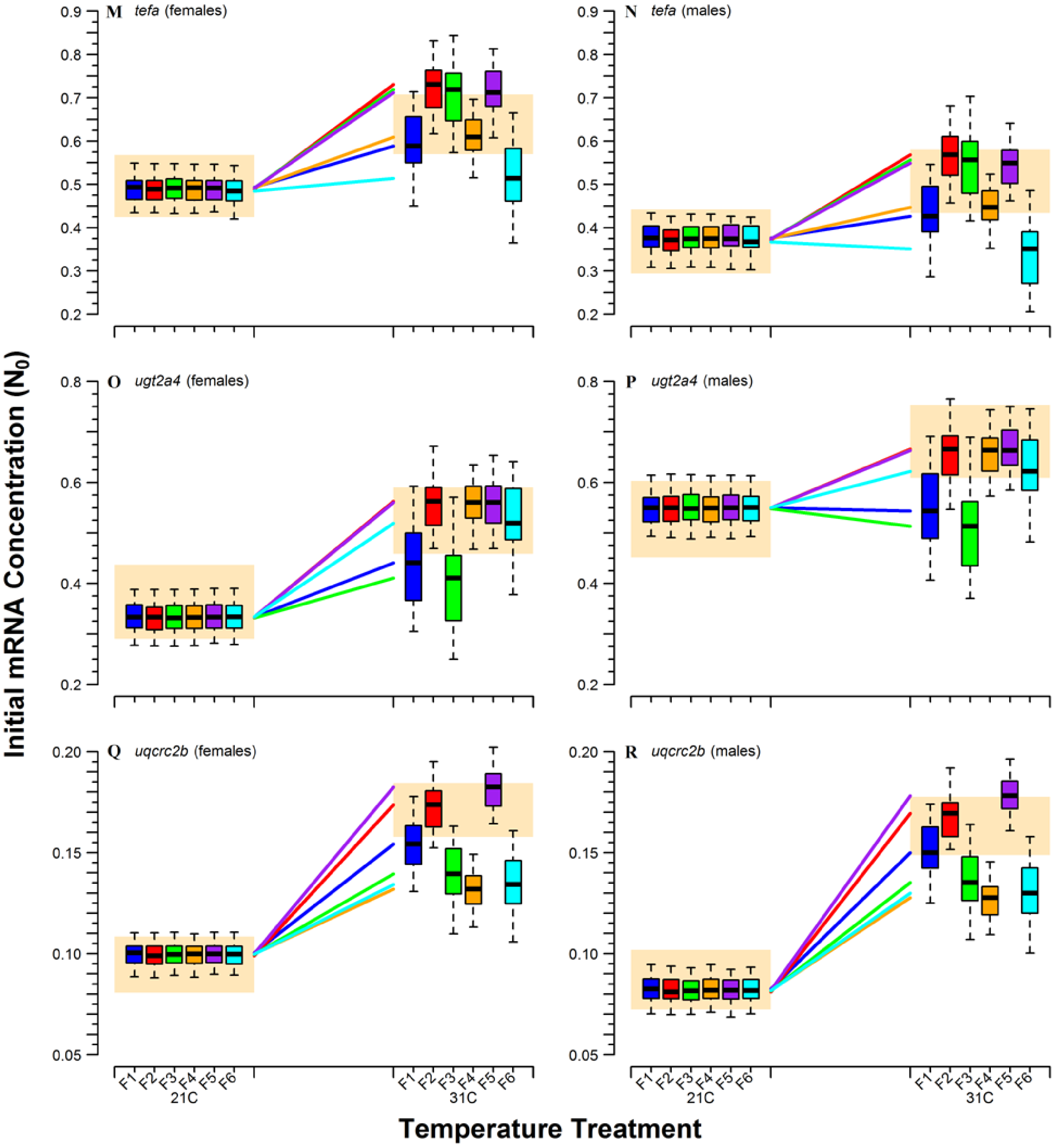

Figure S2. Family-specific reaction norms for genes with non-significant or marginal/questionable G×E for expression in the rainbowfish *Melanotaenia duboulayi*. Plotting as per Figure S1.

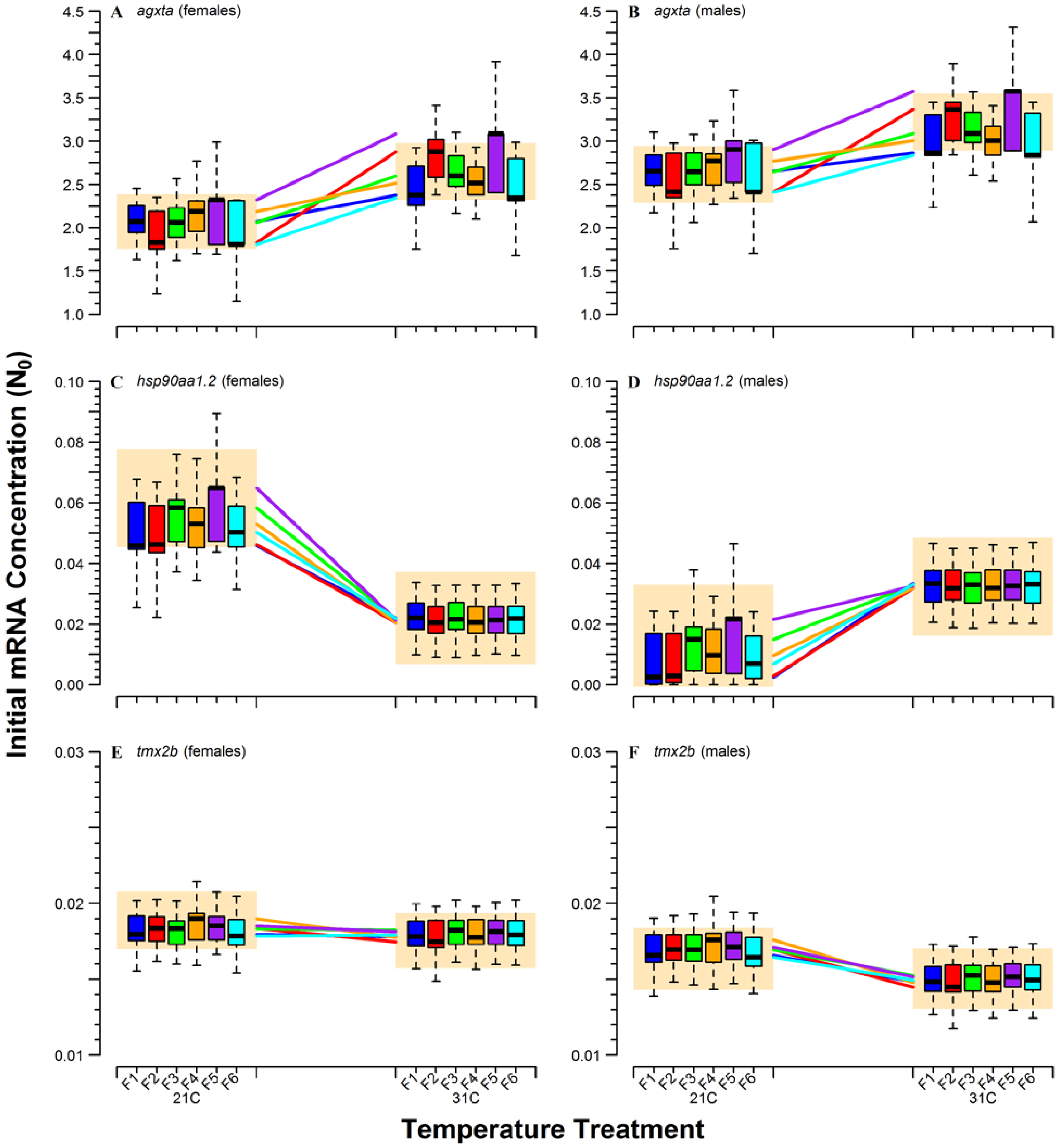

Figure S3. Interactions between proteins for the hsp90aa1.2 sub-network. Black lines with terminal dots denote a biochemical reaction; purple lines with a terminal dot denote catalysis; pink lines with a terminal dot indicate a post-translational modification; green lines and/or terminal arrows indicate an induction/activation; and blue lines denote binding.

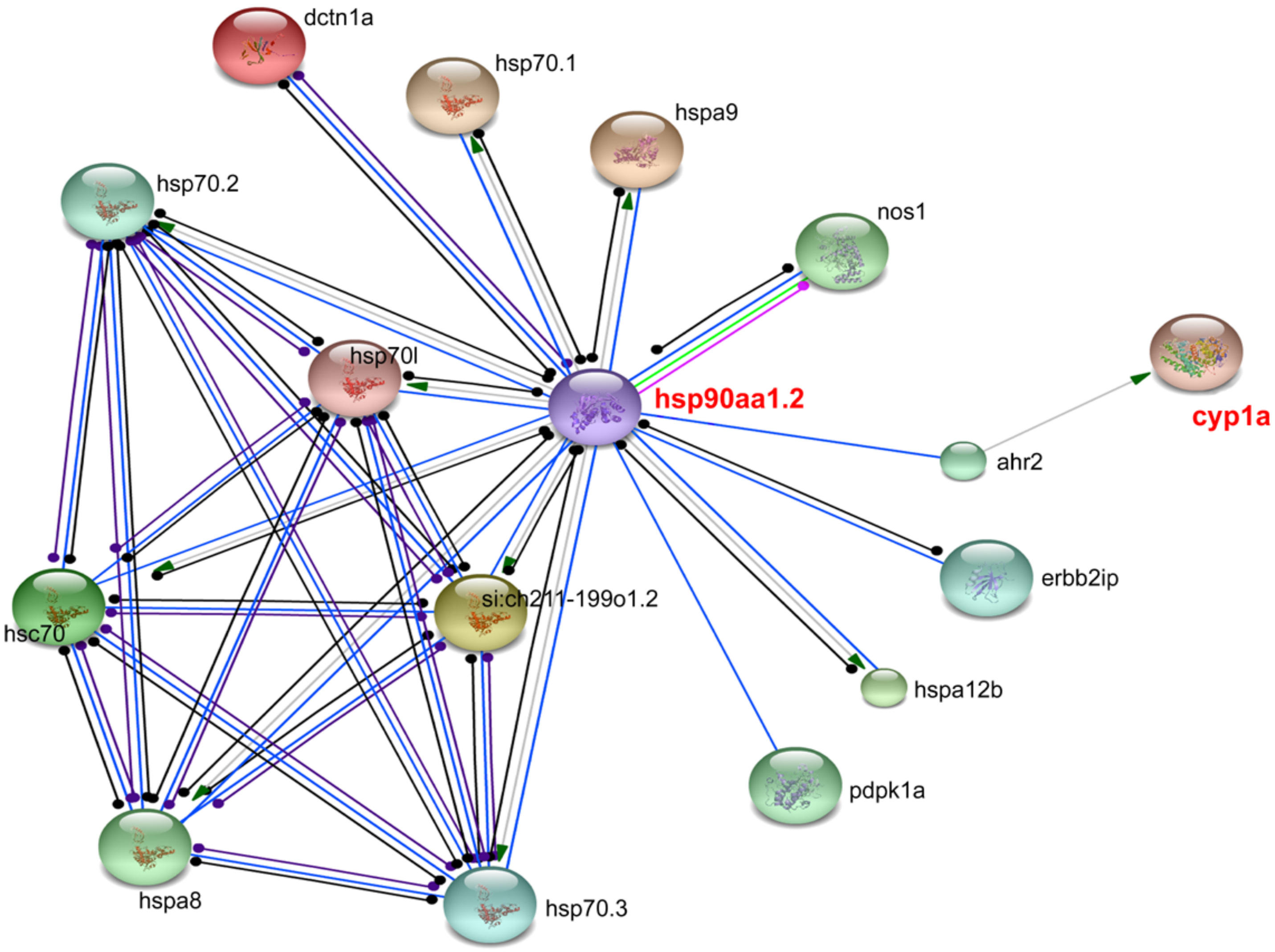

Figure S4. Interactions between proteins for the pparab/hmgcs1 sub-network. Black lines with terminal dots denote a biochemical reaction; yellow lines with a terminal dot indicate evidence of co-expression; green lines and/or terminal arrows indicate an induction/activation; and blue lines denote binding.

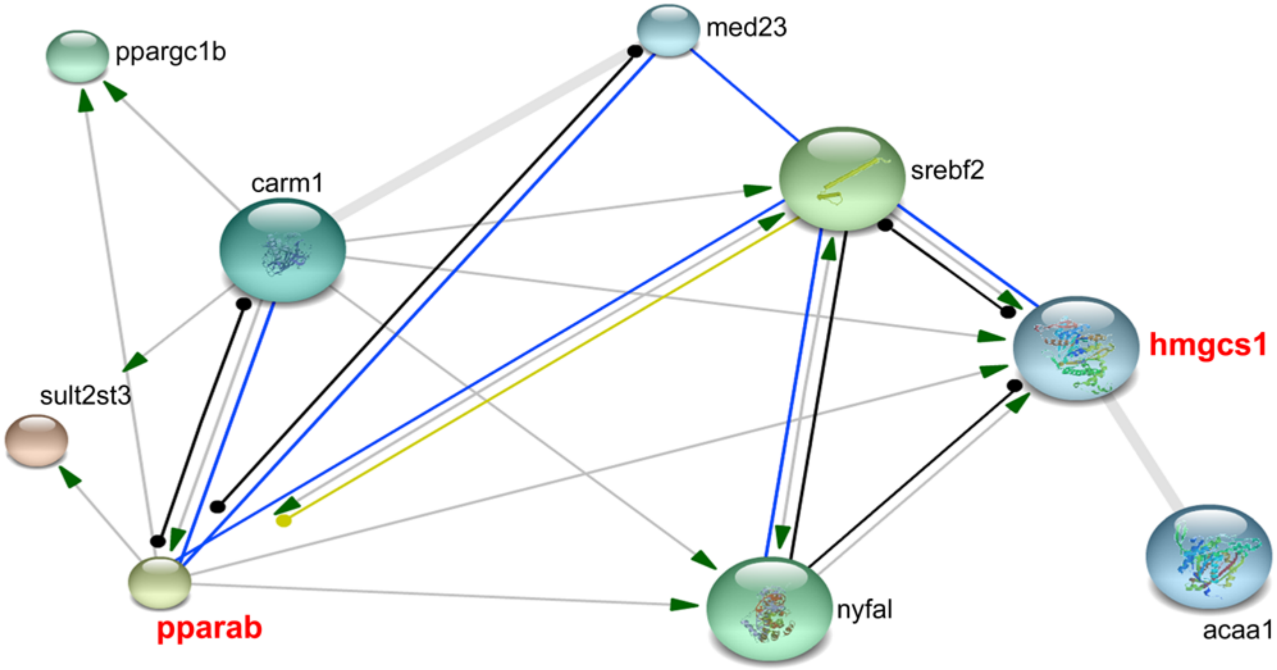

Supplement: Supplementary file 1 — Table S1. Primer sequences of reference and candidate genes. Table S2 Putative protein family identity and functional role(s) of candidate genes in the rainbowfish Melanotaenia duboulayi. Table S3. Variance estimates from nested mixed‐effects models in the rainbowfish Melanotaenia duboulayi. Figure S1. Family‐specific reaction norms for genes with significant G × E for expression in the rainbowfish Melanotaenia duboulayi. Figure S2. Family‐specific reaction norms for genes with non‐significant or marginal/questionable G × E for expression in the rainbowfish Melanotaenia duboulayi. Figure S3. Interactions between proteins for the hsp90aa1.2 sub‐network. Figure S4. Interactions between proteins for the pparab/hmgcs1 sub‐network. [file EVA-9-531-s001.pdf]
